# MEOWA-KTC: A New Distance Measure for Random Permutation Sets Based on MEOWA Weights and Kendall’s Tau Coefficient

**DOI:** 10.3390/e28090970

**Published:** 2026-08-31

**Authors:** Chengyi Jin, Luyuan Chen, Hao Li

**Affiliations:** College of Information Science and Technology & Artificial Intelligence, Nanjing Forestry University, Nanjing 210037, China; jinchengyi@njfu.edu.cn (C.J.); lh1997@njust.edu.cn (H.L.)

**Keywords:** distance measure, information fusion, random permutation set, Kendall’s tau coefficient, MEOWA

## Abstract

Distance measures in random permutation set (RPS) theory are crucial for characterizing inconsistency among permutation-based information distributions. However, existing RPS discrepancy measures do not explicitly distinguish ordering conflicts according to their positional importance under propensity semantics. To address this issue, this paper proposes a new RPS distance, termed MEOWA-KTC, by combining maximum-entropy-based ordered weighted averaging (MEOWA) weights with Kendall’s tau coefficient (KTC). Specifically, MEOWA-KTC constructs a top-weighted similarity between permutation events by using KTC to evaluate the ordinal consistency of corresponding sub-permutations and MEOWA weights controlled by an adjustable orness parameter to emphasize discrepancies at leading positions. Additionally, a spectral correction is applied to ensure that the proposed distance satisfies the metric axioms. Numerical examples and ablation results demonstrate the positional sensitivity of the proposed distance and the respective contributions of MEOWA weighting and KTC. Based on this distance, a fusion model is further developed to derive source support degrees and fusion weights from pairwise RPS distances. In the threat-assessment application, the proposed method produces stable decisions and generally larger decision margins than the benchmark methods. Monte Carlo experiments further demonstrate its robustness to mass-distribution and permutation-order noise.

## 1. Introduction

Real-world information is often characterized by various forms of uncertainty, such as fuzziness, imprecision, ignorance, and incompleteness. In many practical decision-making problems, uncertain assessments also encode rankings among candidate hypotheses based on preference, plausibility, or priority. This type of order-structured uncertainty commonly arises in multi-sensor target and threat assessment, risk prioritization, fault diagnosis, and multi-expert decision-making. Its effective representation and processing are therefore essential for uncertainty modeling and reliable decision analysis. To address diverse forms of uncertain information, various theoretical frameworks have been developed, including probability theory [1], possibility theory [2], Dempster–Shafer evidence theory (evidence theory, DST) [3,4], Z-number theory [5] and fuzzy set theory [6,7]. Among them, DST has garnered significant attention for its ability to reason effectively under imprecise information, making it particularly valuable in scenarios with constrained prior knowledge [8,9,10]. Building upon the theoretical foundation of DST, a number of extended frameworks have been developed to model more complex forms of uncertainty. For example, D numbers relax the mutually exclusive assumption of the classical frame of discernment and are therefore suitable for representing evidence defined on non-exclusive hypotheses [11,12]. Quantum evidence theory extends classical evidence theory by incorporating quantum probability amplitudes and quantum-state representations, thereby enabling uncertainty modeling through quantum superposition and interference effects [13,14,15]. In addition, Random Permutation Set (RPS) theory has been proposed by extending the combination space of basic events to a permutation space, thereby significantly broadening its representational capability in addressing order-structured uncertain information [16,17].

RPS theory characterizes ordered information through event permutations, and defines the permutation mass function and orthogonal rule to quantify the occurrence possibility of propositions and to combine propositions, respectively. As a core feature of RPS theory, the semantic interpretation of ordered information is crucial for understanding information distribution and for supporting both theoretical development and practical applications. Since this issue is not explicitly clarified in the original formulation of RPS, Zhou et al. further examined the meaning of order from the perspectives of random finite sets (RFS) [18] and the transferable belief model (TBM) [19]. In the RFS-based view, order is treated as an additional random attribute beyond the type and cardinality of elements, whereas in the TBM-based view, order is interpreted as qualitative propensity information, indicating the decision-maker’s belief transfer tendency, where elements in leading positions imply stronger preference or higher credibility than those in lower positions. Based on this interpretation, Zhou et al. further proposed Layer-2 Dempster’s combination rule for fusing different RPSs [19] and OWA-based probability transformation algorithm for decision making [20]. This TBM-based semantic view has also motivated subsequent studies on RPS theory, for instance, the RPS construction method based on statistical characteristics [21] and the ordered credal C-means clustering method [22]. Following this line of research, this paper also investigates RPS from the TBM perspective, namely the propensity semantics of ordered information.

Discrepancy and similarity measures provide fundamental tools for quantifying differences and agreement among information distributions, thereby supporting conflict detection, source credibility evaluation, fusion-weight determination, and reliable decision-making under uncertainty [23]. Within the RPS framework, Chen et al. [24] first proposed an RPS distance by incorporating the ordered degree of permutation events into the weighting matrix of the Jousselme distance [25]. Subsequently, the Permutation Jensen–Shannon (PJS) divergence was developed by extending the Jensen–Shannon divergence to RPSs, providing an information-theoretic measure of the discrepancy between permutation mass functions [26]. Chen and Cai [27] further proposed the symmetric Rényi–Permutation (SRP) divergence, which incorporates the structural uncertainty of permutation events into a symmetric Rényi-based formulation and has been applied to conflict management and reliability assessment. Collectively, these studies have enriched RPS discrepancy measurement through quadratic-form and information-theoretic formulations and demonstrated its utility in conflict management and reliability assessment. Nevertheless, the positional semantics of ordering remain insufficiently addressed under the TBM-based interpretation of RPSs. Specifically, the distance proposed by Chen et al. does not explicitly distinguish discrepancies occurring at different ranking positions, whereas PJS and SRP primarily characterize the global divergence between permutation mass distributions. This limitation is particularly important because, under the TBM-based interpretation, order information in an RPS reflects qualitative propensity. Elements appearing in leading positions are associated with stronger preference or higher credibility than those in tail positions. Accordingly, discrepancies involving top-ranked elements should contribute more to the distance than those occurring only among low-ranked elements. However, existing RPS discrepancy measures do not explicitly incorporate such positional semantics and may therefore fail to accurately capture discrepancy variations induced by different element propensities.

To address the above issues, this paper proposes a new MEOWA-KTC distance measure for RPS theory by combining maximum-entropy-based ordered weighted averaging (MEOWA) weights with Kendall’s tau coefficient (KTC). Specifically, a local similarity is constructed at each prefix depth, with KTC evaluating the ordinal consistency between corresponding sub-permutations. These depth-wise similarities are then aggregated using MEOWA weights controlled by an adjustable orness parameter to obtain a top-weighted similarity between permutation events, with greater emphasis on discrepancies at leading positions. The corresponding global similarity matrix is subsequently corrected through spectral decomposition so that the resulting MEOWA-KTC distance satisfies the metric axioms. Furthermore, a distance-based fusion model is developed by transforming pairwise RPS distances into similarity-based support degrees and fusion weights. Numerical examples and a threat assessment application validate the rationality and effectiveness of the proposed method.

The main contributions of this paper are threefold. First, a MEOWA-KTC distance measure is proposed for RPSs by embedding a top-weighted permutation-event similarity matrix into a quadratic-form distance framework. Second, an orness-controlled top-weighted mechanism is introduced to aggregate sub-permutation similarities with MEOWA weights, thereby reflecting the propensity semantics of order information and assigning larger penalties to discrepancies involving top-ranked elements. Third, a distance-based fusion model is developed for RPS-based information fusion and decision analysis, with its effectiveness verified through numerical examples and a threat assessment application.

The rest of this paper is organized as follows. Section 2 introduces some related preliminaries. Section 3 introduces the proposed MEOWA-KTC measure. Section 4 uses several numerical examples to illustrate MEOWA-KTC. Section 5 provides an application for threat assessment and Section 6 concludes our paper.

## 2. Preliminaries

### 2.1. Dempster–Shafer Evidence Theory

Evidence theory is an efficient framework for representing and reasoning with uncertainty, and has become an important tool for uncertainty modeling in various complex systems [28,29,30]. It has been widely applied to information fusion [31], pattern classification [32,33], target recognition [34,35], emergency rescue [36], and risk assessment [37]. Some basic concepts in evidence theory are summarized as follows.

**Definition** **1** (Frame of discernment)**.** *Let Θ be the Frame of Discernment (FOD), which consists of a fixed set of N mutually exclusive and exhaustive elements, denoted by [38,39]*

(1) $$\Theta =\{\theta_{1},\theta_{2},\ldots ,\theta_{N}\}$$

PS(Θ) is the power set of Θ, which contains $2^{N}$ possible subsets, indicated by

(2) $$\begin{array}{cc} PS(\Theta )= & \left\{A_{1},A_{2},\cdots ,A_{2^{N}}\right\}\\ = & \{⌀,\left\{\theta_{1}\right\},\left\{\theta_{2}\right\},\cdots ,\left\{\theta_{N}\right\},\\ \; & \left\{\theta_{1},\theta_{2}\right\},\left\{\theta_{1},\theta_{3}\right\},\cdots ,\left\{\theta_{1},\theta_{N}\right\},\cdots ,\Theta \} \end{array}$$

**Definition** **2** (Mass function)**.** *The mass function, also referred to as the basic probability assignment (BPA), assigns a value between 0 and 1 to each subset (proposition) within $2^{\Theta }$, formally defined by [38,39]*

(3) m:PS(Θ)→[0,1]

*constrained by*

(4) $$\sum_{A\in PS(\Theta )}m\left(A\right)=1,\;\;\;m\left(\emptyset \right)=0$$

*A* is called a focal element if m(A)>0.

**Definition** **3** (Combination rules)**.** *Given two BPAs defined on the same PS(*Θ*), Dempster’s rule of combination is defined by [38,39]*

(5) $$m\left(A\right)=\left\{\begin{array}{cc} \frac{1}{1-K}\sum_{B\cap C=A}m_{1}\left(B\right)m_{2}\left(C\right), & A\neq \emptyset \\ 0, & A=\emptyset \end{array}\right.$$

*where the conflict coefficient K is defined as*

(6) $$K=\underset{B\cap C=\emptyset }{\Sigma }m_{1}\left(B\right)m_{2}\left(C\right)$$

### 2.2. Random Permutation Set Theory

In many real-world decision-making problems, information is often presented in an ordered form and therefore requires appropriate modeling tools. RPS theory is therefore introduced to represent and process order-structured uncertainty, with its theoretical foundation rooted in the physical interpretation of Deng entropy [40,41,42]. Building on this foundation, existing studies on RPS theory have mainly evolved along three research directions, including construction methods of RPS [43], information measurement of ordered information [27,44], and combination rules for multi-source information [45]. Some fundamental definitions of RPS theory are as follows:

#### 2.2.1. Basic Representations

**Definition** **4** (Permutation event space)**.** *Given the FOD *Θ* of N mutually exclusive and exhaustive objects $\theta_{i}$ where i= 1, 2, …, N, its Permutation Event Space (PES) is a set of all possible permutations of *Θ*, denoted as PES(*Θ*) [46].*

(7) $$\begin{array}{ccc} PES\left(\Theta \right) & = & \left\{\;A_{ij}\;|\;i=0,\ldots ,N;\;j=1,\ldots ,P\left(N,i\right)\;\right\}\\ & = & \{\;\;\emptyset ,\left(\theta_{1}\right),\left(\theta_{2}\right),\ldots ,\left(\theta_{N}\right),\left(\theta_{1},\theta_{2}\right),\left(\theta_{2},\theta_{1}\right),\ldots ,\left(\theta_{N-1},\theta_{N}\right),\\ & & \left(\theta_{N},\theta_{N-1}\right),\ldots ,\left(\theta_{1},\theta_{2},\ldots ,\theta_{N}\right),\ldots ,\left(\theta_{N},\theta_{N-1},\ldots ,\theta_{1}\right)\;\;\} \end{array}$$

$P\left(N,i\right)=\frac{N!}{\left(N-i\right)!}$, which represents the *i*-permutation of *N*.

**Definition** **5** (Permutation mass function)**.** *A permutation mass function (PMF), denoted by M, is a rule that assigns a value between 0 and 1 to every ordered set in PES(Θ). This assignment must satisfy the following conditions [46]:*

(8) M:PES(Θ)→[0,1]

*satisfying*

(9) $$M\left(\emptyset \right)=0,\;\;\sum_{A\in PES(\Theta )}M(A)=1$$

*RPS is a set of pairs defined as follows:*

(10) RPS(Θ)={<A,M(A)>|A∈PES(Θ)}

Additionally, combination rules for fusing different RPSs have also been developed, although they are not elaborated here. These studies indicate that RPS theory has established a basic mathematical framework for representing and reasoning with order-structured uncertainty.

#### 2.2.2. Distance Measure for RPS Theory

To quantify the distance between two RPSs, Chen et al. extended the classical Jousselme distance in evidence theory to the permutation space, thereby first proposing the RPS distance measure. Specifically, given two RPSs, $RPS_{1}$ and $RPS_{2}$, defined on the same Θ, their distance is defined as [24]:

(11) $$d_{Chen}\left(RPS_{1},RPS_{2}\right)\;=\;\sqrt{\frac{1}{2}\left(\vec{M_{1}}\;-\;\vec{M_{2}}\right)\underline{RD}{\left(\vec{M_{1}}\;-\;\vec{M_{2}}\right)}^{T}}$$

where $\vec{M_{1}}$ and $\vec{M_{2}}$ are two vectors in the permutation event space with coordinates M(A). $\underline{RD}$ is a similarity matrix whose elements are

(12) $$\underline{RD}\left(A,B\right)=\frac{\left|A\;\;\vec{\cap }\;\;B\right|}{\left|A\;\;\vec{\cup }\;\;B\right|}\times \exp\left(-\frac{\sum_{Y\in A\cap B}\left|{\mathrm{rank}}_{A}\left(Y\right)-{\mathrm{rank}}_{B}\left(Y\right)\right|}{\left|A\cup B\right|}\right)$$

where $rank_{A}$ and $rank_{B}$ represent the position of *Y* in permutation events A and B.

#### 2.2.3. Layer-2 Dempster’s Combination Rule

In the original RPS literature, the left (right) orthogonal fusion rule was proposed for evidence updating [46]. However, this combination rule suffers from the drawback of losing the ordered information from one of the sources. To address this issue, Zhou et al. proposed a new fusion method from the propensity-based perspective [19]. They interpreted RPS as a layer-2 belief structure. Specifically, the layer-1 structure is represented by a BPA over the power set, while the layer-2 structure is represented by an RPS over the permutation set. Based on this model, they developed the layer-2 Dempster’s combination rule (l2-DCR) for RPS fusion, as summarized in Algorithm 1.
**Algorithm 1:** Layer-2 Dempster’s combination rule [19]
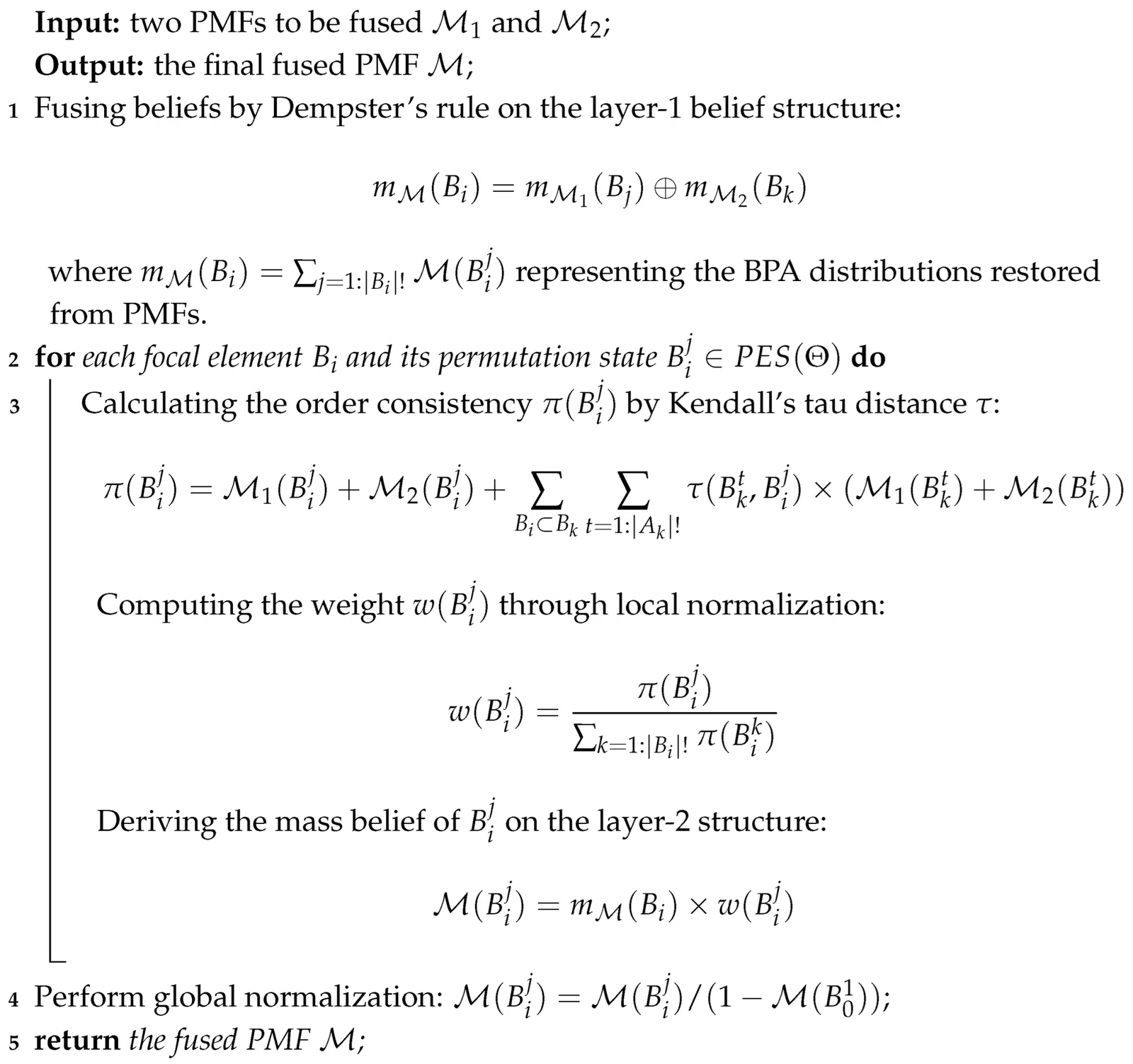


### 2.3. Maximum-Entropy-Based OWA Weight

The ordered weighted averaging (OWA) operator assigns weights to the ordered positions of input values [47]. In the maximum-entropy-based OWA (MEOWA) model, the weight vector is obtained by maximizing entropy under a prescribed orness degree α, which provides a minimally biased weighting scheme consistent with the desired aggregation attitude.

Let $\omega =(\omega_{1},\omega_{2},\ldots ,\omega_{n})$ be the OWA weight vector, where $\omega_{i}$ corresponds to the *i*-th largest ordered value. The MEOWA weights are determined by

(13) $$\max_{\omega }\;H\left(\omega \right)=-\sum_{i=1}^{n}\omega_{i}\ln\omega_{i},$$

subject to

(14) $$\frac{\sum_{i=1}^{n}\left(n-i\right)\omega_{i}}{n-1}=\alpha ,\;\sum_{i=1}^{n}\omega_{i}=1,\;0\leq \omega_{i}\leq 1.$$

According to the analytical solution derived by Filev and Yager [48], the MEOWA weights can be expressed as

(15) $$\omega_{i}^{\left[\alpha \right]}=\frac{h^{n-i}}{\sum_{j=1}^{n}h^{n-j}},\;i=1,2,\ldots ,n,$$

where *h* is the unique positive solution of

(16) $$\sum_{i=1}^{n}\left(\frac{n-i}{n-1}-\alpha \right)h^{n-i}=0.$$

For α∈[0.5,1], the MEOWA weight vector satisfies

(17) $$\omega_{1}^{\left[\alpha \right]}\geq \omega_{2}^{\left[\alpha \right]}\geq \cdots \geq \omega_{n}^{\left[\alpha \right]},$$

which means that higher-ranked elements are assigned weights no smaller than lower-ranked elements. Therefore, MEOWA provides a natural position-dependent weighting mechanism for ranking-related elements under the maximum-entropy principle.

### 2.4. Kendall’s Tau Coefficient

Given two rankings *S* and *T* over the same set of *n* items, Kendall’s Tau Coefficient (KTC), commonly denoted as τ, is used to measure their ordinal agreement. It compares all $\frac{1}{2}n\left(n-1\right)$ item pairs and counts the numbers of concordant and discordant pairs. A pair is concordant if the two rankings assign the same relative order to the two items, and discordant if the relative order is reversed. KTC is defined as [49]

(18) $$\begin{array}{c} \tau \left(S,T\right)=\frac{C-D}{\frac{1}{2}n\left(n-1\right)} \end{array}$$

where *C* and *D* are the numbers of concordant and discordant pairs, respectively. The coefficient satisfies τ∈[−1,1], with τ=1 representing complete agreement, τ=−1 representing complete disagreement, and τ=0 indicating no ordinal correlation.

## 3. MEOWA-KTC: The Distance Measure for RPS Based on MEOWA Weight and Kendall’s Tau Coefficient

In this section, we first examine the positional-discrimination limitations of $d_{Chen}$ [24], PJS [26], and SRP [27] using a numerical example, and then introduce the proposed MEOWA-KTC measure.

### 3.1. Counterintuitive Behavior of Existing Measures

Existing RPS discrepancy measures characterize differences between permutation mass functions from different perspectives. However, they may not distinguish ordering conflicts according to the ranking positions at which they occur, thereby overlooking their different implications under propensity semantics. Example 1 illustrates this issue.

**Example** **1.** *Given the frame of discernment $\Theta =\{\theta_{1},\theta_{2},\theta_{3}\}$, three RPSs are defined as follows:*

$$\begin{array}{cc} \; & RPS_{1}=\left\{\langle \left(\theta_{1},\theta_{2},\theta_{3}\right),1\rangle \right\},\\ \; & RPS_{2}=\left\{\langle \left(\theta_{1},\theta_{3},\theta_{2}\right),1\rangle \right\},\\ \; & RPS_{3}=\left\{\langle \left(\theta_{2},\theta_{1},\theta_{3}\right),1\rangle \right\}. \end{array}$$

For a broader comparison, the pairwise discrepancies among the three RPSs are evaluated using $d_{Chen}$, PJS, and SRP. The resulting values are $d_{Chen}\left(RPS_{1},RPS_{2}\right)=d_{Chen}\left(RPS_{1},RPS_{3}\right)=0.6976$ and $d_{Chen}\left(RPS_{2},RPS_{3}\right)=0.8581$. PJS assigns a value of 1.0000 to all three pairs, while SRP assigns a value of 16.6096 to all three pairs. The SRP calculation uses the zero-mass replacement $\varepsilon =10^{-5}$ in this study. Because these measures have different definitions and numerical ranges, the comparison focuses on their ability to distinguish different types of ordering conflict rather than on their absolute values.

Taking $RPS_{1}$ as the reference, $RPS_{2}$ preserves the leading element $\theta_{1}$ and changes only the order of the two lower-ranked elements, whereas $RPS_{3}$ changes the leading element. Therefore, under the propensity semantics of RPSs, the discrepancy between $RPS_{1}$ and $RPS_{3}$ should be larger than that between $RPS_{1}$ and $RPS_{2}$. Nevertheless, $d_{Chen}$ assigns the same value to these two pairs. Neither PJS nor SRP distinguishes these two types of conflict. Since the three RPSs are point masses on distinct complete permutation events, both divergences treat each comparison as involving two distinct event atoms without evaluating the ranking positions at which their internal orders differ. These observations motivate a distance measure that explicitly accounts for positional propensity.

### 3.2. The Proposed MEOWA-KTC Measure for RPSs

Motivated by the counterintuitive behavior discussed in Section 3.1, this section develops a new RPS distance by constructing a top-weighted similarity between permutation events. The proposed method is designed to preserve the propensity semantics conveyed by the ordinal information of permutation events. Specifically, its construction considers two aspects. First, the ordinal consistency between corresponding sub-permutation events should be evaluated to characterize the agreement between their internal orders. Second, similarities obtained at different prefix depths should receive different levels of importance so that discrepancies involving leading elements have a greater influence. Accordingly, KTC is used to quantify ordinal consistency at each prefix depth, and MEOWA weights are used to aggregate the resulting similarities with position-dependent importance. The resulting top-weighted permutation-event similarity is then embedded into a quadratic-form distance framework to measure inconsistency between RPSs. The formal definitions follow.

**Definition** **6** (Similarity measure between permutation events)**.** *Let A and B be two permutation events in PES(Θ), their similarity S(A,B) is calculated by aggregating the weighted similarity among all sub-permutation events:*

(19) $$Sim\left(A,B\right)=\sum_{d=1}^{L}\omega_{d}^{\alpha }\;\chi \left(A_{d},B_{d},d\right)$$

where L=max(|A|,|B|) denotes the maximum length of the two permutations, and $\omega_{d}^{\alpha }$ is the MEOWA weight assigned to $\chi (A_{d},B_{d},d)$, namely, the local similarity of the *d*-th sub-permutation event under the orness degree α, as defined by

(20) $$\chi \left(A_{d},B_{d},d\right)=\{\begin{array}{cc} 0, & |A_{d}\cap B_{d}|=0\\ 1/|A_{d}\cup B_{d}|, & |A_{d}\cap B_{d}|=1\\ {\left(\sqrt{|A_{d}\cup B_{d}|}\right)}^{\tau (A_{d},B_{d})-1}, & |A_{d}\cap B_{d}|>1 \end{array}$$

Here, $A_{d}$ and $B_{d}$ denote the sub-permutation events consisting of the first *d* elements of *A* and *B*, respectively. Note that $A_{d}=A$ if d>|A|, and $B_{d}=B$ if d>|B|. When $A_{d}$ and $B_{d}$ have no common element, their similarity $\chi (A_{d},B_{d},d)$ is set to zero. When they share only one common element, $\chi (A_{d},B_{d},d)$ reduces to the Jaccard similarity coefficient because only the structural overlap between the two sub-permutation events needs to be considered. When they share two or more common elements, $\chi (A_{d},B_{d},d)$ further incorporates the relative order of the common elements through $\tau (A_{d},B_{d})$, thereby capturing both structural overlap and ordinal consistency. Letting $m=|A_{d}\cup B_{d}|$, the third case can be written as $\chi \left(A_{d},B_{d},d\right)=m^{[\tau \left(A_{d},B_{d}\right)-1]/2}$. Since $\tau \left(A_{d},B_{d}\right)\in \left[-1,1\right]$, the resulting similarity satisfies $1/m\leq \chi (A_{d},B_{d},d)\leq 1$. In particular, when the common elements occur in perfectly reverse order, $\tau (A_{d},B_{d})=-1$ and the similarity becomes 1/m. This positive lower bound is intentional because the two sub-permutations still share common elements despite their opposite orders, whereas zero similarity is reserved for the cformat, same as below highlight.ase without any common element. Moreover, since 2≤m≤N, the similarity is bounded below by 1/N. Therefore, the perfectly reversed case does not introduce numerical instability and requires no separate treatment. In addition, the orness parameter α in the proposed measure is restricted to [0.5,1]. Over this interval, the MEOWA weights satisfy $\omega_{1}^{\left[\alpha \right]}\geq \omega_{2}^{\left[\alpha \right]}\geq \cdots \geq \omega_{L}^{\left[\alpha \right]}$ (as shown in Equation (17)). Consequently, the local similarities at shallower prefix depths receive no less weight than those at deeper depths, giving the resulting permutation-event similarity its top-weighted property.

To construct an RPS distance from the above permutation-event similarity, a global similarity matrix is first formed. For a fixed frame of discernment Θ and orness degree α, let $E_{1},E_{2},\ldots ,E_{M}$ denote all nonempty permutation events in PES(Θ), where *M* is their total number. The empty event is omitted because its permutation mass is always zero. The global MEOWA-KTC similarity matrix is defined as

$$S^{\left[\alpha \right]}={\left[\mathrm{Sim}\left(E_{i},E_{j}\right)\right]}_{M\times M}.$$

The difference between any two valid permutation mass vectors belongs to the zero-sum subspace. Therefore, the quadratic-form distance depends only on the restriction of $S^{\left[\alpha \right]}$ to this subspace. Although $S^{\left[\alpha \right]}$ is symmetric, it is not necessarily positive semidefinite when restricted to the zero-sum subspace. A spectral correction is therefore performed before constructing the proposed distance.

Let $Q\in R^{M\times (M-1)}$ be a matrix whose columns form an orthonormal basis of the zero-sum subspace, satisfying

(21) $$Q^{T}Q=I_{M-1},\;Q^{T}1=0.$$

Using this basis, $S^{\left[\alpha \right]}$ is projected onto the zero-sum subspace. Since the resulting matrix $B^{\left[\alpha \right]}$ is generally dense, a dense eigendecomposition is performed as

(22) $$B^{\left[\alpha \right]}=Q^{T}S^{\left[\alpha \right]}Q=U\Lambda U^{T},$$

where $\Lambda =\mathrm{diag}(\lambda_{1},\ldots ,\lambda_{M-1})$ contains the eigenvalues and U contains the corresponding orthonormal eigenvectors.

For a small constant ε>0, each eigenvalue is corrected according to ${\tilde{\lambda }}_{i}=\max\left\{\lambda_{i},\varepsilon \right\}$. The corrected matrix is then reconstructed in the projected coordinates as

(23) $${\tilde{B}}^{\left[\alpha \right]}=U\mathrm{diag}\left({\tilde{\lambda }}_{1},\ldots ,{\tilde{\lambda }}_{M-1}\right)U^{T}.$$

In this study, $\varepsilon =10^{-8}$ is used only as a numerical regularization constant.

Finally, the corrected matrix is mapped back to the original permutation-event space and normalized as

(24) $$G^{\left[\alpha \right]}=\frac{Q{\tilde{B}}^{\left[\alpha \right]}Q^{T}}{\lambda_{\max}\left({\tilde{B}}^{\left[\alpha \right]}\right)}.$$

By construction, $G^{\left[\alpha \right]}$ is positive definite on the zero-sum subspace, and its largest eigenvalue on this subspace is one.

**Definition** **7** (The proposed MEOWA-KTC distance for RPSs)**.** *Given two RPSs, $RPS_{1}$ and $RPS_{2}$, defined on the same frame of discernment, their distance calculated by MEOWA-KTC distance is defined as*

(25) $$d_{MK}\left(RPS_{1},RPS_{2}\right)=\sqrt{\frac{1}{2}\left(\vec{M_{1}}-\vec{M_{2}}\right)G^{\left[\alpha \right]}{\left(\vec{M_{1}}-\vec{M_{2}}\right)}^{T}}.$$

*Here, $\vec{M_{1}}$ and $\vec{M_{2}}$ are the permutation mass vectors arranged according to the same complete list of nonempty permutation events. Events that do not occur in a given RPS are assigned zero mass.*

**Proposition** **1** (Metric properties)**.** *For fixed *Θ*, α, and ε>0, the MEOWA-KTC distance in Equation (25) satisfies non-negativity, identity of indiscernibles, symmetry, and the triangle inequality. Moreover, $0\leq d_{MK}\left(RPS_{1},RPS_{2}\right)\leq 1$.*

**Proof.**  Let $\delta_{12}={\left(\vec{M_{1}}-\vec{M_{2}}\right)}^{T}$. Since both permutation mass vectors sum to one, $1^{T}\delta_{12}=0$. Thus, $\delta_{12}$ belongs to the zero-sum subspace and can be written as $\delta_{12}=Qz_{12}$ for some $z_{12}$. Let $c=\lambda_{\max}\left({\tilde{B}}^{\left[\alpha \right]}\right)$. Substituting Equation (24) into Equation (25) gives

(26) $$d_{MK}\left(RPS_{1},RPS_{2}\right)=\frac{1}{\sqrt{2c}}{∥{\left({\tilde{B}}^{\left[\alpha \right]}\right)}^{1/2}Q^{T}\delta_{12}∥}_{2}.$$

Non-negativity follows directly from the non-negativity of the Euclidean norm. Since ${\tilde{B}}^{\left[\alpha \right]}$ is positive definite, Equation (26) equals zero if and only if $Q^{T}\delta_{12}=0$. Because $\delta_{12}$ belongs to the column space of Q, this condition holds if and only if $\delta_{12}=0$, which is equivalent to $RPS_{1}=RPS_{2}$. Symmetry follows because exchanging the two RPSs changes only the sign of $\delta_{12}$.

For any third RPS, $\delta_{13}=\delta_{12}+\delta_{23}$. Applying the Minkowski inequality to Equation (26) gives

$$d_{MK}\left(RPS_{1},RPS_{3}\right)\leq d_{MK}\left(RPS_{1},RPS_{2}\right)+d_{MK}\left(RPS_{2},RPS_{3}\right).$$

Finally, every eigenvalue of ${\tilde{B}}^{\left[\alpha \right]}/c$ lies in (0,1]. Since $\delta_{12}$ belongs to the zero-sum subspace, $∥Q^{T}\delta_{12}{∥}_{2}={∥\delta_{12}∥}_{2}$. Therefore,

$$d_{MK}^{\;2}\left(RPS_{1},RPS_{2}\right)\leq \frac{1}{2}{∥\delta_{12}∥}_{2}^{2}\leq 1,$$

where the last inequality follows because the Euclidean distance between two probability vectors is at most $\sqrt{2}$. Hence, $d_{MK}\left(RPS_{1},RPS_{2}\right)\leq 1$. □

### 3.3. Practical Selection of the Orness Parameter

The orness degree α controls the strength of the top-weighted preference in MEOWA-KTC. When α=0.5, the MEOWA weights are uniform, corresponding to neutral treatment of different prefix depths. As α increases, more weight is assigned to shallower prefixes, thereby increasing the emphasis on leading positions. At the boundary α=1, all weight is assigned to the first prefix depth and the remaining order information is ignored. Therefore, the interval [0.5,0.9] is used in the numerical examples and application experiments to represent neutral to strongly top-weighted preferences while avoiding this degenerate case.

In practical applications, α can be selected according to the required attention depth. Let *d* denote the number of leading positions regarded as important and let κ denote the desired cumulative weight assigned to these positions. For each candidate value in A⊆[0.5,0.9], the corresponding MEOWA weight vector is generated using Equations (15) and (16). An appropriate value of α can then be determined by

(27) $$\alpha^{*}=\underset{\alpha \in A}{\arg\;\min}\left|\sum_{i=1}^{d}\omega_{i}^{\left[\alpha \right]}-\kappa \right|.$$

This strategy converts the intuitive requirement that the top *d* positions should account for approximately κ of the total importance into a specific orness degree. For example, when a decision problem mainly concerns the first two ranking positions, *d* can be set to 2, while κ can be specified according to their desired importance. When reliable quantitative information about *d* and κ is unavailable, α=0.8 can be used as a practical default. This value represents a strong but non-extreme preference for leading positions while retaining the contributions of lower-ranked positions. It is therefore suitable as a reference setting under the usual propensity semantics of RPSs, although it is not claimed to be universally optimal.

### 3.4. Computational Complexity and Scalability Analysis

Let $M_{N}=\sum_{k=1}^{N}P\left(N,k\right)$ denote the number of nonempty permutation events in an *N*-element frame, which is also the dimension of the global similarity matrix. Since $M_{N}=N!\sum_{j=0}^{N-1}1/j!=\Theta \left(N!\right)$, the event-space dimension grows factorially with *N*. The MEOWA weight vectors for different prefix lengths can be precomputed and reused, and their computational cost is of lower order than that of constructing the global similarity matrix.

With direct pair counting, computing KTC at prefix depth *ℓ* requires at most $O(\ell^{2})$ time. Summing this cost over at most *N* prefix depths requires $O(N^{3})$ time for each pair of permutation events. Therefore, constructing the complete similarity matrix requires $O(M_{N}^{2}N^{3})$ time. The projection and dense eigendecomposition specified in Equation (22), together with the reconstruction operations in Equations (23) and (24), require $O(M_{N}^{3})$ time in total. When the prefix-depth similarities are accumulated sequentially, the one-time construction complexities for fixed Θ, α, and ε are

(28) $$T_{\mathrm{con}}=O\left(M_{N}^{2}N^{3}+M_{N}^{3}\right),\;S_{\mathrm{con}}=O\left(M_{N}^{2}\right).$$

Here, $T_{\mathrm{con}}$ and $S_{\mathrm{con}}$ denote the construction time and space complexities, respectively.

Once the corrected global matrix has been constructed, direct evaluation of the quadratic form requires $O(M_{N}^{2})$ time for each pair of RPSs. Since $M_{N}=\Theta \left(N!\right)$ and the $O(M_{N}^{3})$ term asymptotically dominates the construction cost, the one-time construction requires $O({\left(N!\right)}^{3})$ time and $O({\left(N!\right)}^{2})$ space, while each subsequent distance evaluation requires $O({\left(N!\right)}^{2})$ time in the worst case. For example, $M_{6}=1956$, whereas $M_{7}=13699$. Therefore, the current exact implementation is practically suitable mainly for small-scale frames, typically with N≤6. For larger frames, the computation may be restricted to a fixed reduced event universe that contains every permutation event allowed to receive positive mass and is shared by all RPSs under comparison. A structure-preserving sparse approximation may also be considered, provided that the corrected operator remains strictly positive definite on the corresponding zero-sum subspace.

## 4. Numerical Examples and Discussion

In this section, several numerical examples are provided to illustrate the proposed MEOWA-KTC measure and compare it with $d_{Chen}$, PJS, and SRP. Since these methods have different definitions and numerical scales, the comparisons focus on their variation trends rather than the absolute magnitudes of their values. For reproducibility, all numerical examples and experiments in this study were implemented in MATLAB R2025b and executed on a computer running Windows 11 Pro with an Intel Core i7-14650HX processor and 64 GB of RAM.

**Example** **2.** *Following Example 1, the distance values of $RPS_{1}$, $RPS_{2}$, and $RPS_{3}$ are further calculated using the proposed MEOWA-KTC method.*

Based on Equations (15) and (16), the three-dimensional MEOWA weight vector with orness degree α=0.8 is obtained as ω=(0.6819,0.2362,0.0819). Set $A=(\theta_{1},\theta_{2},\theta_{3})$ and $B=(\theta_{1},\theta_{3},\theta_{2})$, Table 1 shows the similarities of their sub-permutations at each depth. Consequently, the total similarity degree between *A* and *B* is computed as follows:

$$\begin{array}{cc} Sim(A,B) & =\sum_{d=1}^{3}\omega_{d}\chi \left(A_{d},B_{d},d\right)=0.6819\times 1+0.2362\times \frac{1}{3}+0.0819\times 0.6934\\ \; & =0.8174 \end{array}$$

Finally, the two deterministic RPSs are embedded into the same complete 15-dimensional permutation event space. Using the corrected global metric matrix $G^{\left[0.8\right]}$, Equation (25) gives

$$\begin{array}{cc} d_{MK}\left(RPS_{1},RPS_{2}\right)\; & =\sqrt{\frac{1}{2}\left(\vec{M_{1}}-\vec{M_{2}}\right)G^{\left[0.8\right]}{\left(\vec{M_{1}}-\vec{M_{2}}\right)}^{T}}\\ \; & =0.2167. \end{array}$$

**Figure 1:**
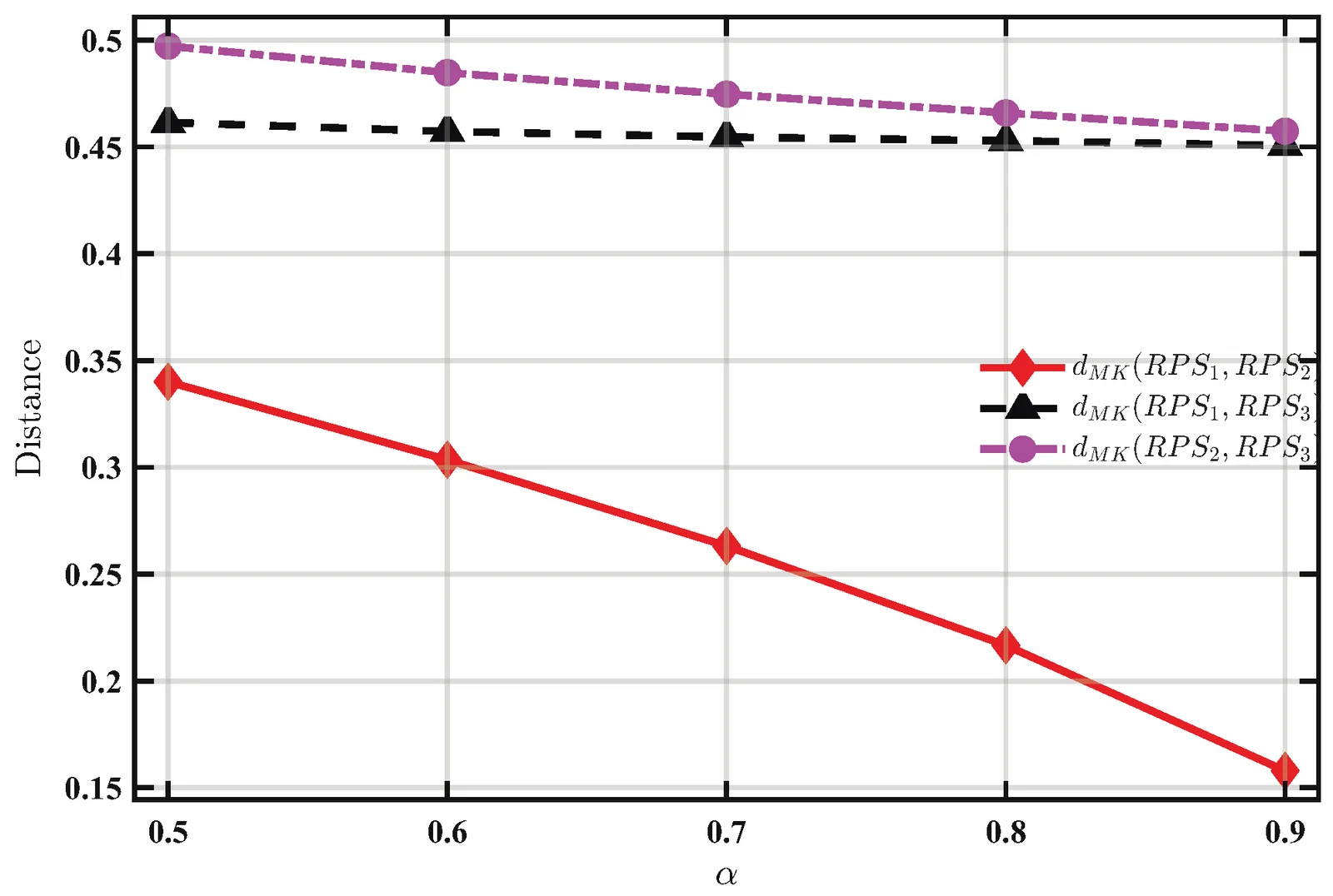
Variation trends of RPS distances with α in Example 1.

For each value of α, a single corrected global matrix is constructed and reused to calculate the three pairwise MEOWA-KTC distances. The results are reported in Table 2 and Figure 1. The ordering $d_{MK}\left(RPS_{1},RPS_{2}\right)<d_{MK}\left(RPS_{1},RPS_{3}\right)<d_{MK}\left(RPS_{2},RPS_{3}\right)$ holds for every tested value of α. In particular, $d_{MK}\left(RPS_{1},RPS_{2}\right)<d_{MK}\left(RPS_{1},RPS_{3}\right)$. By comparison, $d_{Chen}$ assigns the same value to these two pairs, while both PJS and SRP assign the same value to all three pairs. Therefore, the proposed distance distinguishes ordering conflicts occurring at different ranking positions. As α increases, all three MEOWA-KTC distances decrease, with a substantially larger reduction observed for $d_{MK}\left(RPS_{1},RPS_{2}\right)$. This pattern is consistent with the increasing emphasis placed on the common leading element of $RPS_{1}$ and $RPS_{2}$, whereas the other two pairs differ at the first position.

**Example** **3.** *Given a fixed set $\Theta =\{\theta_{1},\theta_{2},\theta_{3}\}$, two RPSs defined on *Θ* are shown as follows.*

$$\begin{array}{cc} \; & RPS_{1}=\left\{<\left(\theta_{2},\theta_{3}\right),1>\right\}\\ \; & RPS_{2}=\left\{<\left(\theta_{1}\right),0.2>,<\left(\theta_{2},\theta_{3}\right),0.1>,<\left(\theta_{1},\theta_{2},\theta_{3}\right),\beta >,<\left(\theta_{3},\theta_{2},\theta_{1}\right),0.7-\beta >\right\} \end{array}$$

**Figure 2:**
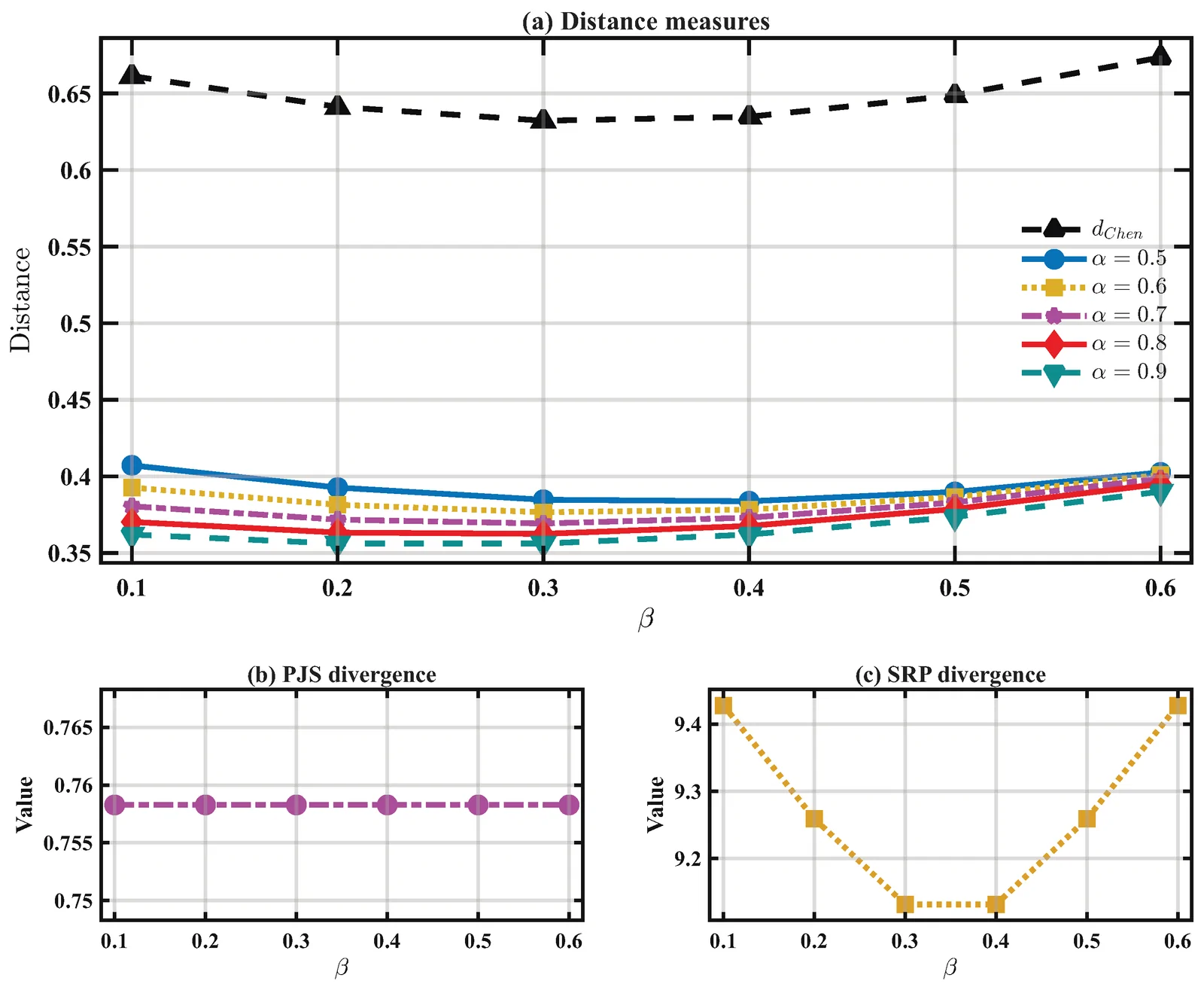
Variation trends of $d_{Chen}$, PJS, SRP, and MEOWA-KTC with β in Example 3, where MEOWA-KTC is evaluated for α=0.5,0.6,0.7,0.8, and 0.9.

In Example 3, the parameter β redistributes the belief mass of $RPS_{2}$ between two permutation events with different element orders. This example is used to examine whether the compared measures reflect variations caused by both belief mass assignments and ordered permutation information. The values obtained by $d_{Chen}$, PJS, SRP, and MEOWA-KTC are reported in Table 3. Figure 2 presents the variation trends of all four measures; separate panels are used because their numerical scales differ substantially.

As shown in Table 3 and Figure 2, $d_{Chen}$, SRP, and all MEOWA-KTC curves vary non-monotonically with β, whereas PJS remains constant. Both $d_{Chen}$ and the MEOWA-KTC distances generally decrease at first and then increase. The minimum MEOWA-KTC distance occurs at an intermediate value of β between 0.2 and 0.4, with its exact location depending on α. PJS remains constant at 0.7583 because $RPS_{1}$ assigns unit mass to $\left(\theta_{2},\theta_{3}\right)$ and $RPS_{2}$ always assigns a mass of 0.1 to the same event. The variation in β only redistributes mass between two permutation events outside the support of $RPS_{1}$, which does not change the PJS divergence in this configuration. SRP responds to this redistribution and exhibits a symmetric pattern, attaining its minimum value of 9.1317 at both β=0.3 and β=0.4. Although SRP responds to changes in the mass distribution, neither PJS nor SRP explicitly evaluates the ordinal similarity between distinct permutation events. For each fixed β, the MEOWA-KTC distance decreases consistently as α increases, demonstrating its sensitivity to changes in positional weighting.

**Example** **4.** *Given a fixed set $\Theta =\{\theta_{1},\theta_{2},\theta_{3},\theta_{4}\}$. Two RPSs defined on *Θ* are shown as follows.*

$$\begin{array}{cc} \; & RPS_{1}=\left\{<\left(\theta_{1}\right),0.8>,<\left(\theta_{2},\theta_{3}\right),0.1>,<\left(X\right),0.1>\right\}\\ \; & RPS_{2}=\left\{<\left(\theta_{1}\right),0.6>,<\left(\theta_{2},\theta_{3}\right),0.2>,<\left(X\right),0.2>\right\} \end{array}$$

*where X denotes a complete permutation of the elements in *Θ*.*

In this example, the two RPSs have the same focal permutation events but different belief mass assignments. Both $RPS_{1}$ and $RPS_{2}$ assign relatively large belief masses to $\left(\theta_{1}\right)$, while the remaining masses are assigned to $\left(\theta_{2},\theta_{3}\right)$ and the complete permutation event *X*. Therefore, this example is used to examine how the distance changes when the internal order of *X* varies. Since this example is intended to isolate the effect of changing the internal order of *X* rather than repeat the preceding parameter analysis, α=0.8 is used as a representative strongly top-weighted but non-degenerate setting.

As shown in Table 4, PJS remains constant at 0.0349, while SRP remains constant at 0.4550 for every choice of *X*. For each *X*, $RPS_{1}$ and $RPS_{2}$ share the same permutation-event support, while their aligned mass vectors remain fixed at (0.8,0.1,0.1) and (0.6,0.2,0.2). Because neither divergence explicitly compares the internal order of a shared support event, their values remain unchanged as *X* varies across different complete permutations. In contrast, both $d_{Chen}$ and $d_{MK}$ incorporate pairwise similarities between permutation events and therefore vary with the internal ordering of *X*. When *X* begins with $\theta_{1}$, the MEOWA-KTC distance ranges from 0.0363 to 0.0382. When $\theta_{1}$ is moved away from the leading position, the distance increases to between 0.0480 and 0.0577, with the largest value obtained for $X=(\theta_{2},\theta_{3},\theta_{4},\theta_{1})$. This clear separation demonstrates the sensitivity of MEOWA-KTC to the position of $\theta_{1}$ within *X*, particularly whether it occupies the leading position.

**Example** **5.** *Given a fixed set $\Theta =\{\theta_{1},\theta_{2},\theta_{3},\theta_{4},\theta_{5},\theta_{6}\}$, two RPSs defined on *Θ* are given as follows:*

$$\begin{array}{cc} \; & RPS_{1}=\left\{\langle \left(\theta_{1},\theta_{2},\theta_{3},\theta_{4},\theta_{5},\theta_{6}\right),0.7\rangle ,\langle \left(\theta_{1}\right),0.3\rangle \right\},\\ \; & RPS_{2}=\left\{\langle \left(A_{k}\right),0.7\rangle ,\langle \left(\theta_{1}\right),0.3\rangle \right\}. \end{array}$$

*where $A_{k}$ denotes the permutation obtained by swapping the elements at the k-th and (k+1)-th positions, as illustrated in Table 5.*

In Example 5, $RPS_{1}$ assigns a belief mass of 0.7 to the original complete permutation $\left(\theta_{1},\theta_{2},\theta_{3},\theta_{4},\theta_{5},\theta_{6}\right)$ and a belief mass of 0.3 to the single-element event $\left(\theta_{1}\right)$. $RPS_{2}$ has the same mass assignment structure, but replaces the original complete permutation with $A_{k}$. Therefore, this example is designed to examine whether the compared measures can distinguish perturbations occurring at different positions.

**Figure 3:**
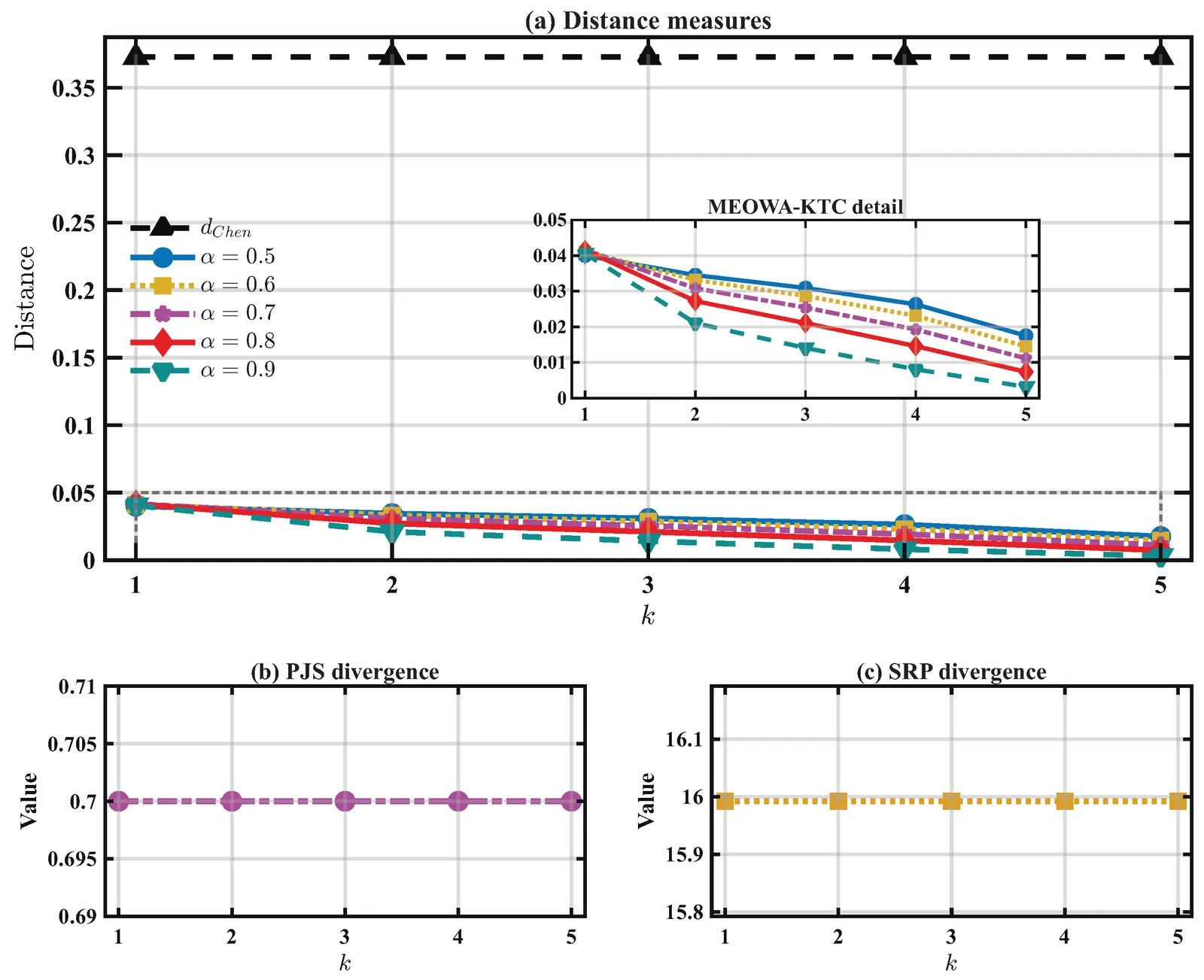
Variation trends of $d_{Chen}$, PJS, SRP, and MEOWA-KTC for different adjacent-swap positions *k* in Example 5. The inset enlarges the MEOWA-KTC curves, while the separate PJS and SRP panels preserve their respective numerical scales.

As shown in Table 6 and Figure 3, $d_{Chen}$, PJS, and SRP remain unchanged across all adjacent-swap positions and therefore appear as horizontal lines. Specifically, $d_{Chen}$ remains constant at 0.3727, showing that it does not distinguish the ranking positions at which the swaps occur. PJS and SRP remain constant at 0.7000 and 15.9922, respectively. For every *k*, $A_{k}$ is a complete permutation of the same length and is distinct from the original complete permutation, while the two RPSs retain the same mass-assignment structure. Consequently, neither PJS nor SRP distinguishes the position of the adjacent swap. In contrast, for every tested value of α, the MEOWA-KTC distance decreases monotonically as *k* increases from 1 to 5. This result indicates that adjacent swaps occurring closer to the leading position produce larger distances in this example. For $A_{2}$–$A_{5}$, the distance also decreases as α increases, which is consistent with the greater weights assigned to their unchanged leading prefixes. The distance for $A_{1}$ remains close to 0.04 across all tested values of α because this swap changes the first-ranked element and preserves no common leading prefix. Therefore, MEOWA-KTC distinguishes adjacent swaps occurring at different ranking positions and exhibits the intended leading-position sensitivity.

To examine the contributions of MEOWA weighting and KTC, the adjacent-swap experiment in Example 5 is repeated using four variants. MEOWA-KTC uses α=0.8, while Uniform-KTC uses uniform weights and is equivalent to MEOWA-KTC with α=0.5. MEOWA-Jaccard retains the MEOWA weights with α=0.8 but replaces KTC with Jaccard similarity. Uniform-Jaccard uses uniform weights and Jaccard similarity. For each variant, a corrected global matrix is constructed and reused for all five comparisons. The positional discrimination ratio is defined as $R_{\mathrm{pos}}=\left[d\left(A_{1}\right)-d\left(A_{5}\right)\right]/d\left(A_{1}\right)$, where a larger value indicates stronger positional discrimination. Here, $d(A_{k})$ denotes the distance between $RPS_{1}$ and the corresponding $RPS_{2}$ constructed using $A_{k}$.

As shown in Table 7, all four variants distinguish adjacent swaps occurring at different positions. With KTC fixed, replacing uniform weighting with MEOWA weighting at α=0.8 increases $R_{\mathrm{pos}}$ from 0.5624 to 0.8234. With Jaccard similarity fixed, the corresponding ratio increases from 0.4204 to 0.8286. These paired comparisons demonstrate that MEOWA weighting substantially strengthens the distinction between perturbations occurring at leading and trailing positions. Under both weighting schemes, the KTC-based variants produce larger distances than their Jaccard-based counterparts at every perturbation position. This result indicates that KTC captures relative-order disagreement that cannot be represented by prefix-set overlap alone. Overall, MEOWA weighting governs the positional emphasis, while KTC characterizes the relative order of common elements.

## 5. Experiment

### 5.1. A New RPS-Based Data Fusion Method

Data fusion aims to integrate data from multiple sources, sensors, or systems to obtain a more accurate and reliable representation of the target object or environment. In practical applications, multi-source data often involve uncertainty as well as ordered information, where the order and position of elements may influence the final fusion result. By using the proposed MEOWA-KTC to evaluate the position-sensitive differences between RPSs, a new RPS-based data fusion method is introduced for uncertain ordered information fusion. The specific fusion process is shown as follows.

Step-1 Given a set of *n* RPSs collected from different sensor reports, compute the pairwise distance $d_{MK}\left(RPS_{i},RPS_{j}\right)$(i,j=1,2,…,n) according to Equation (25).
Step-2 Convert the distance between $RPS_{i}$ and $RPS_{j}$ into a similarity measure as follows:
  (29) $$Sim\left(RPS_{i},RPS_{j}\right)=1-d_{MK}\left(RPS_{i},RPS_{j}\right).$$
Step-3 Determine the support degree of each RPS $RPS_{i}$, which reflects the overall consensus received from the other evidence sources:
  (30) $$Sup\left(RPS_{i}\right)=\sum_{j=1,j\neq i}^{n}Sim\left(RPS_{i},RPS_{j}\right).$$
Step-4 Obtain the credibility degree of each RPS by normalizing its support degree:
  (31) $$Cre\left(RPS_{i}\right)=\frac{Sup(RPS_{i})}{\sum_{j=1}^{n}Sup\left(RPS_{j}\right)}.$$
Step-5 Construct the weighted RPS $RPS^{\omega }$ by assigning credibility weights to the original RPSs:
  (32) $$RPS^{\omega }=\sum_{i=1}^{n}Cre\left(RPS_{i}\right)RPS_{i}.$$
Step-6 Based on the Layer-2 Dempster’s combination rule, combine $RPS^{\omega }$ with itself (n−1) times to generate the final fused evidence $RPS^{f}$:
  (33) $$RPS^{f}={\left(\ldots {\left({\left(RPS^{\omega }⊕RPS^{\omega }\right)}_{1}⊕RPS^{\omega }\right)}_{2}⊕\cdots ⊕RPS^{\omega }\right)}_{n-1}.$$

### 5.2. Experiment Results for Threat Assessment

This section investigates the effectiveness of the proposed MEOWA-KTC-based fusion method through an enemy aircraft threat assessment problem. The frame of discernment is given by $\Theta =\{\theta_{1},\theta_{2},\theta_{3}\}$, representing three potential targets. In this scenario, radar reports are modeled as RPSs, where each permutation event characterizes a possible threat-ranking pattern and its corresponding mass indicates the assigned belief degree. The assessment task is to determine the most plausible threat event under different event cardinalities, including Type-1 events, Type-2 events and Type-3 events. Unlike conventional single-target assessment, this problem requires evaluating not only individual targets but also ordered target combinations with different cardinalities. Following Deng et al. [46], the relative threat degree (RTD) is adopted to support such multi-target assessment by locally normalizing the fused mass over permutation events with the same cardinality:

(34) $$RTD\left(A_{ij}\right)=\frac{M(A_{ij})}{\sum_{j=1}^{P(N,i)}M\left(A_{ij}\right)},\;i=1,2,3,\;j=1,\ldots ,P\left(3,i\right),$$

where $A_{ij}$ denotes the *j*-th threat event containing *i* targets, and $M(A_{ij})$ is the fused mass assigned to $A_{ij}$. Accordingly, the final assessment result for each event cardinality is obtained by selecting the event with the maximum RTD:

(35) $$A_{i}^{*}=\arg\max_{1\leq j\leq P(N,i)}RTD\left(A_{ij}\right),\;i=1,\ldots ,3.$$

The original radar reports used in this case study are presented in Table 8. As shown in this table, $RPS_{1}$ mainly provides the detailed ranking $\left(\theta_{1},\theta_{3},\theta_{2}\right)$, whereas $RPS_{3}$ mainly provides the coarse report $\left(\theta_{1}\right)$. Thus, the two reports differ substantially in granularity but agree that $\theta_{1}$ is the leading threat. $RPS_{2}$ is an abnormal conflicting report that assigns its main masses to $\left(\theta_{2}\right)$ and rankings beginning with $\theta_{2}$. The expected decisions are fixed before comparison as $\left(\theta_{1}\right)$, $\left(\theta_{1},\theta_{2}\right)$, and $\left(\theta_{1},\theta_{3},\theta_{2}\right)$ for Type-1, Type-2, and Type-3 events, respectively.

To broaden the experimental comparison, the threat assessment is conducted using $d_{Chen}$, PJS, SRP, and the proposed MEOWA-KTC distance. The fusion procedure based on PJS follows the reliability assessment framework proposed in [26]. The same procedure is adopted for SRP, with the PJS divergence replaced by the SRP divergence. After the source credibilities have been obtained, all methods follow the same remaining steps.

As shown in Table 9, all benchmark methods and MEOWA-KTC settings identify the same dominant threat events, namely $\left(\theta_{1}\right)$ for Type-1, $\left(\theta_{1},\theta_{2}\right)$ for Type-2, and $\left(\theta_{1},\theta_{3},\theta_{2}\right)$ for Type-3. This agreement shows that MEOWA-KTC produces final decisions consistent with those of the benchmark methods in this application. For Type-2 and Type-3, the dominant-event RTD values obtained using MEOWA-KTC are higher than those obtained using $d_{Chen}$, PJS, and SRP for every tested value of α. For Type-1, MEOWA-KTC produces higher values than $d_{Chen}$ and SRP throughout the tested range. It also exceeds PJS when α≥0.7 and remains close to PJS when α=0.5 and 0.6. Thus, MEOWA-KTC preserves the final event selections while generally producing higher RTD values for the selected events.

To further quantify the separation between the selected event and its strongest competitor, the decision margin is defined as $\Delta_{\mathrm{RTD}}=RTD_{\left(1\right)}-RTD_{\left(2\right)}$, where $RTD_{\left(1\right)}$ and $RTD_{\left(2\right)}$ denote the largest and second-largest RTD values within the same event type, respectively. The resulting decision margins are presented in Table 10.

As shown in Table 10, MEOWA-KTC produces larger decision margins than the traditional $d_{Chen}$ method for all three threat-event types and every tested value of α. For Type-2 and Type-3, its margins also exceed those obtained using PJS and SRP throughout the tested range. For Type-1, MEOWA-KTC exceeds SRP for every value of α and exceeds PJS when α≥0.7, while remaining close to PJS when α=0.5 and 0.6. Moreover, the decision margins increase monotonically with α for all three threat-event types, reaching 0.5577, 0.2956, and 0.3629, respectively, at α=0.9. In this application, this trend indicates that increasing α enlarges the separation between the dominant threat event and its strongest competitor. Therefore, MEOWA-KTC maintains stable final decisions while generally improving the discriminability of the assessment results in this scenario involving a conflict at the leading position.

### 5.3. Robustness Analysis Under Noise Perturbations

To evaluate the robustness of threat assessment to imperfect RPS inputs, two Monte Carlo experiments are conducted using the radar reports in Table 8. MEOWA-KTC with α=0.8 is compared with $d_{Chen}$, PJS, and SRP, with the same credibility-weighted fusion and RTD-based decision procedures applied to all methods. The two experiments introduce random perturbations into the permutation mass distributions and internal permutation orders, respectively, while preserving the validity of the resulting RPSs. Robustness is evaluated using the decision stability rate and the decision margin. The decision stability rate is the proportion of trials in which the pre-specified dominant event is recovered for each threat-event type, while the decision margin measures the separation between the dominant and second-ranked events.

The first experiment evaluates robustness to perturbations in the permutation mass distributions. For source *s*, let $\vec{M_{s}}$ denote its original permutation mass vector. At noise level η, the perturbed permutation mass vector is generated as

(36) $$\vec{{\tilde{M}}_{s}}=\left(1-\eta \right)\vec{M_{s}}+\eta \vec{U_{s}},$$

where $\vec{U_{s}}$ is an independently sampled random permutation mass vector that follows the uniform distribution over the probability simplex defined by all 15 nonempty permutation events in PES(Θ). The tested noise levels are η∈{0,0.05,0.10,0.15,0.20,0.30}. Because both $\vec{M_{s}}$ and $\vec{U_{s}}$ are valid permutation mass vectors, their convex combination remains nonnegative and normalized. Increasing η replaces a larger proportion of the original permutation mass distribution with random information and therefore represents a stronger level of mass-distribution noise.

**Figure 4:**
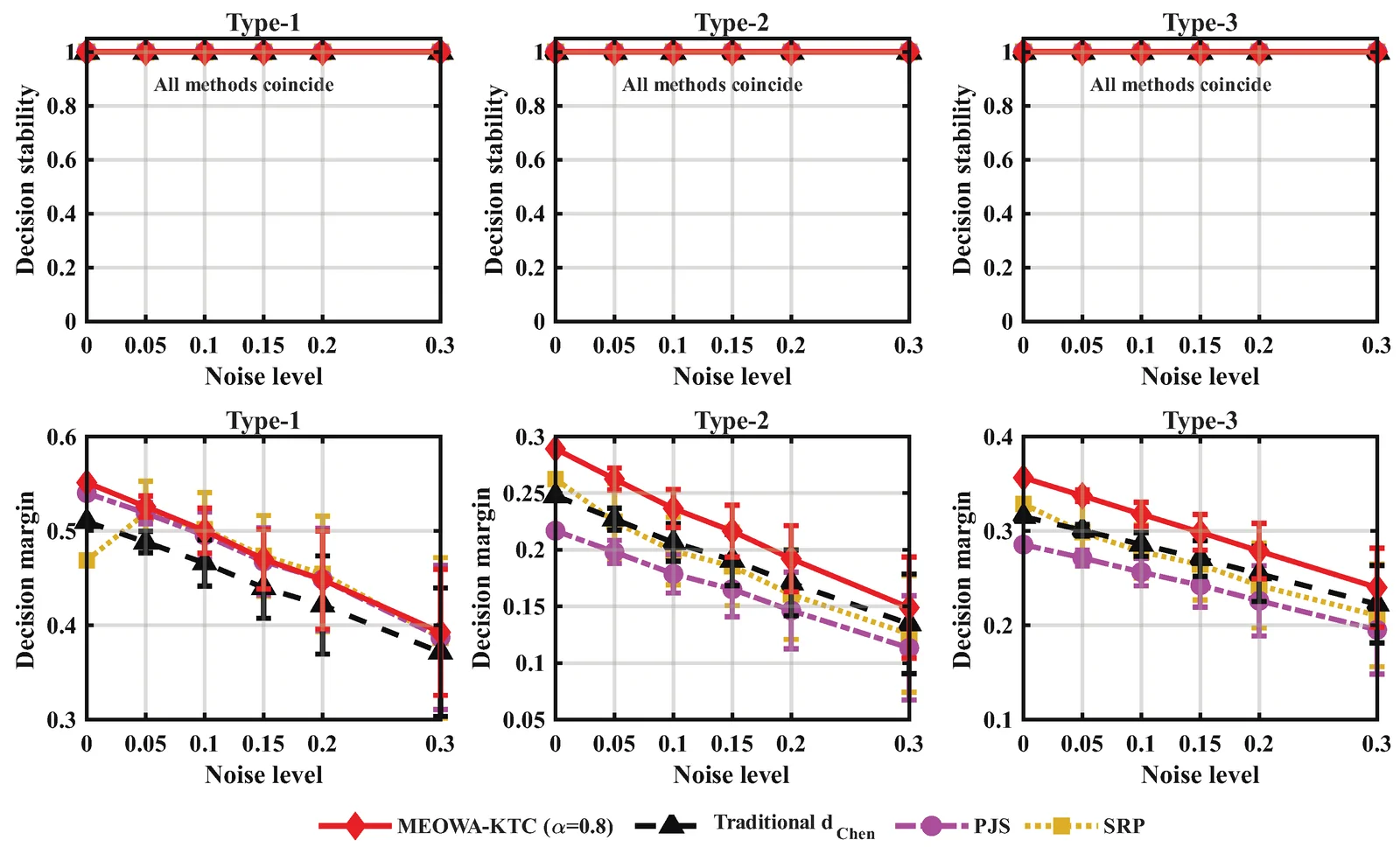
Robustness under mass-distribution noise. The upper row shows decision stability, and the lower row shows the mean decision margin with SD error bars.

For each value of η, 100 independent trials are conducted using a fixed random seed of 2026. Within each trial, all four methods are evaluated using exactly the same perturbed RPS samples to ensure a paired comparison. Figure 4 reports the decision stability rates and mean decision margins with standard deviation error bars over the 100 trials. As shown in Figure 4, all four methods maintain a decision stability rate of 1.0000 for all three threat-event categories throughout the tested range of η. This result indicates that the selected dominant events remain unchanged under the tested levels of mass-distribution noise. Nevertheless, the mean decision margins generally decrease as η increases. For Type-1, MEOWA-KTC, $d_{Chen}$, and PJS show overall decreasing trends, whereas SRP initially increases slightly and then declines. For Type-2 and Type-3, all four methods exhibit steady decreases, with MEOWA-KTC maintaining the largest mean decision margin at every noise level. At η=0.30, MEOWA-KTC also produces the largest mean decision margin for Type-1. Although the standard deviations generally increase with the noise level, the proposed method retains stable event selections and comparatively strong decision separation under the tested mass-distribution perturbations.

**Figure 5:**
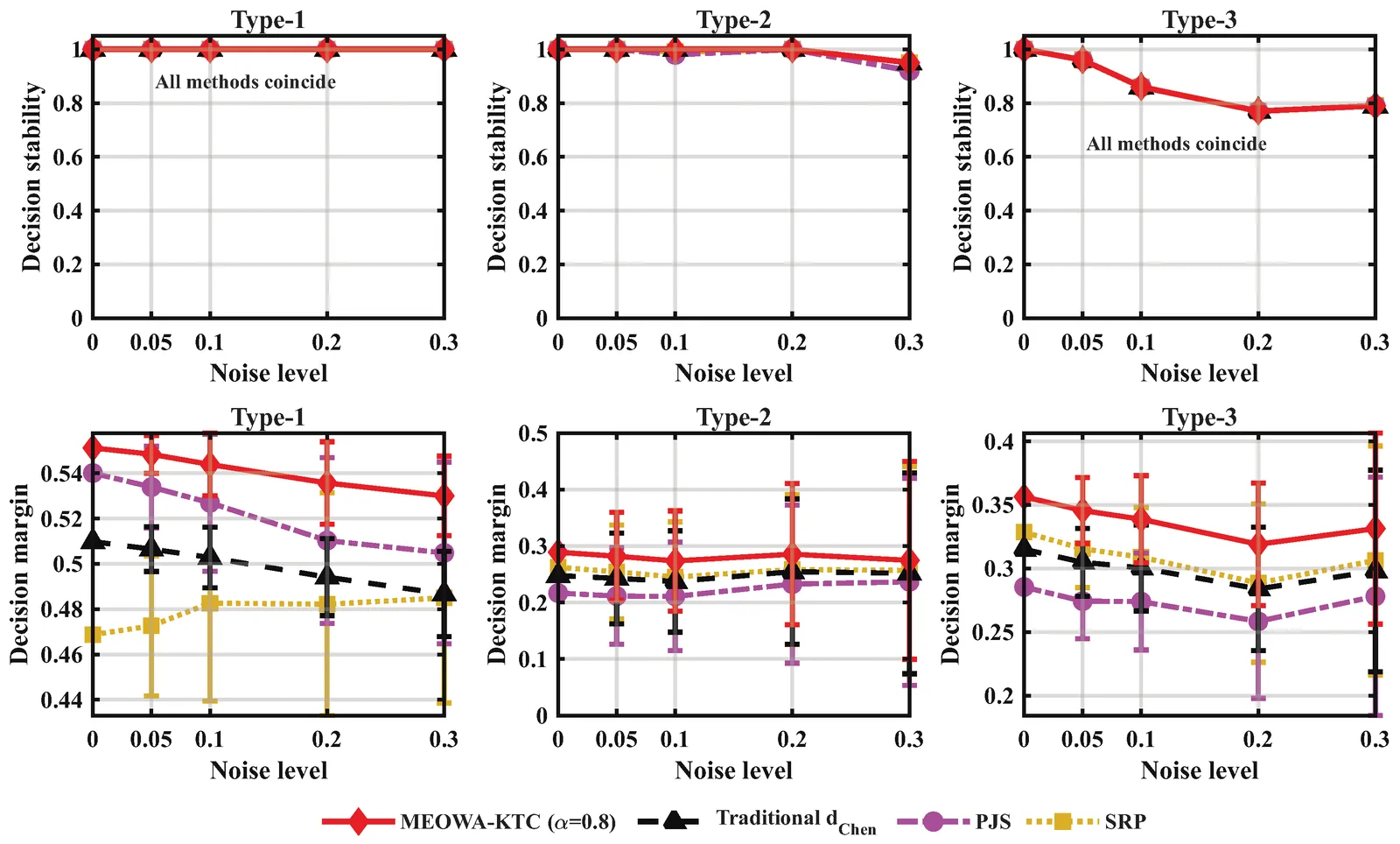
Robustness under permutation-order noise. The upper row shows decision stability, and the lower row shows the mean decision margin with SD error bars.

The second experiment evaluates robustness to perturbations in the internal orders of permutation events. At noise level ρ, each non-singleton permutation event with positive mass in a source RPS is independently perturbed with probability ρ. When an event is selected, a pair of adjacent elements is chosen uniformly at random and swapped, and the mass of the original event is transferred to the corresponding reordered event. Events that are not selected retain their original orders and masses. The tested noise levels are ρ∈{0,0.05,0.10,0.20,0.30}. This procedure preserves the total permutation mass while directly perturbing the ordinal information represented by the RPSs. For each value of ρ, 100 independent trials are conducted using a fixed random seed of 2026. Within each trial, all four methods are evaluated using exactly the same perturbed RPS samples. Figure 5 reports the resulting decision stability rates and mean decision margins with standard deviation error bars.

As shown in Figure 5, the effects of permutation-order noise differ across the three threat-event categories. For Type-1, all four methods maintain a decision stability rate of 1.0000 throughout the tested range. Type-2 also remains highly stable. At ρ=0.30, the stability rates are 0.9500 for MEOWA-KTC, $d_{Chen}$, and SRP and 0.9200 for PJS. For Type-3, all four methods produce identical stability rates, which decrease to 0.7700 at ρ=0.20 and then increase slightly to 0.7900 at ρ=0.30. Regarding the decision margins, MEOWA-KTC maintains the largest mean value for every threat-event category and noise level. For Type-1, the mean margins of MEOWA-KTC, $d_{Chen}$, and PJS gradually decrease, whereas SRP shows a slight overall increase. For Type-2, the margins initially decrease, increase at ρ=0.20, and then decline slightly at ρ=0.30. For Type-3, the margins decrease until ρ=0.20 and then increase at ρ=0.30. At the maximum tested noise level, MEOWA-KTC produces the largest mean decision margin for all three threat-event categories.

To facilitate a quantitative comparison at the maximum tested noise levels, Table 11 summarizes the decision stability rates and decision margins obtained at η=0.30 and ρ=0.30.

As shown in Table 11, MEOWA-KTC produces the largest mean decision margin for all three threat-event categories under both noise models. Its decision stability rates are equal to or higher than those of the benchmark methods. These endpoint results are consistent with the overall trends observed in Figure 4 and Figure 5. Under mass-distribution noise, all four methods preserve the dominant-event selections, although their decision margins decrease as the noise level increases. Permutation-order noise has a greater effect on the more detailed Type-3 decisions, whose stability rate decreases to 0.79 for all methods at ρ=0.30. Nevertheless, MEOWA-KTC retains the largest mean decision margins under both noise models. These results support its robustness to the tested perturbations in RPS inputs.

## 6. Conclusions

This paper proposed a MEOWA-KTC distance measure for RPSs to characterize inconsistency in order-structured uncertain information. Unlike existing RPS discrepancy measures, MEOWA-KTC incorporates both ordinal consistency and position-dependent importance. KTC evaluates the ordinal consistency between corresponding sub-permutations, while MEOWA weights controlled by an adjustable orness degree α assign greater importance to leading positions. This construction enables the proposed distance to reflect the propensity semantics of RPSs by placing greater emphasis on discrepancies involving top-ranked elements. A spectral correction was further applied to ensure that the resulting distance satisfies the metric axioms. Based on the proposed distance, an RPS fusion model was developed in which source credibilities are derived from pairwise RPS similarities and used in evidence fusion. The relative threat degrees are then calculated to support decision-making in the threat-assessment application.

Numerical examples demonstrate that the proposed distance distinguishes ordering conflicts occurring at different ranking positions. The ablation results further verify the complementary contributions of MEOWA weighting and KTC, with the former governing positional emphasis and the latter capturing the relative order of common elements. In the threat-assessment application, all methods identify the same dominant events, while MEOWA-KTC generally produces larger decision margins than $d_{Chen}$, PJS, and SRP, particularly at larger values of α. Monte Carlo experiments under mass-distribution and permutation-order noise show that MEOWA-KTC maintains decision stability comparable to or higher than that of the benchmark methods. It also generally produces larger mean decision margins and achieves the largest values for all three threat-event categories at the maximum tested noise levels. These results support the effectiveness and robustness of MEOWA-KTC in the examined scenarios.

Nevertheless, these findings should not be interpreted as a universal guarantee for arbitrary RPS data. The factorial growth of the complete permutation-event space restricts the current exact implementation to small-scale frames. Developing scalable approximation strategies that preserve the metric properties of MEOWA-KTC therefore remains an important direction for future research. 

## Figures and Tables

**Table 1:** The similarity of sub-permutations of A and B at different depth.

Depthd	$A_{d}$	$B_{d}$	$\chi (A_{d},B_{d},d)$
1	$\left(\theta_{1}\right)$	$\left(\theta_{1}\right)$	1
2	$\left(\theta_{1},\theta_{2}\right)$	$\left(\theta_{1},\theta_{3}\right)$	1/3
3	$\left(\theta_{1},\theta_{2},\theta_{3}\right)$	$\left(\theta_{1},\theta_{3},\theta_{2}\right)$	0.6934

**Table 2:** RPS distances under different orness parameters α.

α	$d_{\mathrm{MK}}$ (${\mathrm{RPS}}_{1}$, ${\mathrm{RPS}}_{2}$)	$d_{\mathrm{MK}}\left({\mathrm{RPS}}_{1},{\mathrm{RPS}}_{3}\right)$	$d_{\mathrm{MK}}\left({\mathrm{RPS}}_{2},{\mathrm{RPS}}_{3}\right)$
0.5	0.3401	0.4614	0.4972
0.6	0.3035	0.4573	0.4848
0.7	0.2632	0.4546	0.4747
0.8	0.2167	0.4529	0.4659
0.9	0.1579	0.4507	0.4574

**Table 3:** Values of the compared measures under different values of α and β in Example 3.

β	$d_{\mathrm{Chen}}$	PJS	SRP	MEOWA-KTC (Under Different α)				
				α=0.5	α=0.6	α=0.7	α=0.8	α=0.9
0.1	0.6614	0.7583	9.4278	0.4072	0.3927	0.3805	0.3703	0.3620
0.2	0.6412	0.7583	9.2592	0.3927	0.3815	0.3718	0.3633	0.3562
0.3	0.6322	0.7583	9.1317	0.3848	0.3766	0.3692	0.3625	0.3562
0.4	0.6348	0.7583	9.1317	0.3838	0.3783	0.3730	0.3677	0.3620
0.5	0.6488	0.7583	9.2592	0.3898	0.3866	0.3830	0.3787	0.3735
0.6	0.6735	0.7583	9.4278	0.4026	0.4010	0.3986	0.3951	0.3901

**Table 4:** Values of the compared measures when *X* changes in Example 4.

*X*	$d_{\mathrm{Chen}}$	PJS	SRP	$d_{\mathrm{MK}}$ (α=0.8)
$\theta_{1},\theta_{2},\theta_{3},\theta_{4}$	0.1674	0.0349	0.4550	0.0382
$\theta_{1},\theta_{2},\theta_{4},\theta_{3}$	0.1654	0.0349	0.4550	0.0376
$\theta_{1},\theta_{3},\theta_{2},\theta_{4}$	0.1674	0.0349	0.4550	0.0372
$\theta_{1},\theta_{3},\theta_{4},\theta_{2}$	0.1654	0.0349	0.4550	0.0372
$\theta_{1},\theta_{4},\theta_{2},\theta_{3}$	0.1638	0.0349	0.4550	0.0365
$\theta_{1},\theta_{4},\theta_{3},\theta_{2}$	0.1638	0.0349	0.4550	0.0363
$\theta_{2},\theta_{1},\theta_{3},\theta_{4}$	0.1732	0.0349	0.4550	0.0542
$\theta_{2},\theta_{1},\theta_{4},\theta_{3}$	0.1707	0.0349	0.4550	0.0539
$\theta_{2},\theta_{3},\theta_{1},\theta_{4}$	0.1788	0.0349	0.4550	0.0573
$\theta_{2},\theta_{3},\theta_{4},\theta_{1}$	0.1807	0.0349	0.4550	0.0577
$\theta_{2},\theta_{4},\theta_{1},\theta_{3}$	0.1732	0.0349	0.4550	0.0558
$\theta_{2},\theta_{4},\theta_{3},\theta_{1}$	0.1776	0.0349	0.4550	0.0565
$\theta_{3},\theta_{1},\theta_{2},\theta_{4}$	0.1687	0.0349	0.4550	0.0487
$\theta_{3},\theta_{1},\theta_{4},\theta_{2}$	0.1672	0.0349	0.4550	0.0486
$\theta_{3},\theta_{2},\theta_{1},\theta_{4}$	0.1732	0.0349	0.4550	0.0511
$\theta_{3},\theta_{2},\theta_{4},\theta_{1}$	0.1751	0.0349	0.4550	0.0516
$\theta_{3},\theta_{4},\theta_{1},\theta_{2}$	0.1697	0.0349	0.4550	0.0508
$\theta_{3},\theta_{4},\theta_{2},\theta_{1}$	0.1732	0.0349	0.4550	0.0512
$\theta_{4},\theta_{1},\theta_{2},\theta_{3}$	0.1672	0.0349	0.4550	0.0482
$\theta_{4},\theta_{1},\theta_{3},\theta_{2}$	0.1672	0.0349	0.4550	0.0480
$\theta_{4},\theta_{2},\theta_{1},\theta_{3}$	0.1713	0.0349	0.4550	0.0511
$\theta_{4},\theta_{2},\theta_{3},\theta_{1}$	0.1751	0.0349	0.4550	0.0520
$\theta_{4},\theta_{3},\theta_{1},\theta_{2}$	0.1713	0.0349	0.4550	0.0508
$\theta_{4},\theta_{3},\theta_{2},\theta_{1}$	0.1751	0.0349	0.4550	0.0513

**Table 5:** Permutations $A_{k}$ obtained by adjacent swaps for k=1,…,5.

*k*	$A_{k}$
1	$\left(\theta_{2},\theta_{1},\theta_{3},\theta_{4},\theta_{5},\theta_{6}\right)$
2	$\left(\theta_{1},\theta_{3},\theta_{2},\theta_{4},\theta_{5},\theta_{6}\right)$
3	$\left(\theta_{1},\theta_{2},\theta_{4},\theta_{3},\theta_{5},\theta_{6}\right)$
4	$\left(\theta_{1},\theta_{2},\theta_{3},\theta_{5},\theta_{4},\theta_{6}\right)$
5	$\left(\theta_{1},\theta_{2},\theta_{3},\theta_{4},\theta_{6},\theta_{5}\right)$

**Table 6:** Values of the compared measures under different values of *k* and α in Example 5.

$A_{k}$	$d_{\mathrm{Chen}}$	PJS	SRP	$d_{\mathrm{MK}}$ Under Different Values of α				
				α=0.5	α=0.6	α=0.7	α=0.8	α=0.9
$A_{1}$	0.3727	0.7000	15.9922	0.0400	0.0411	0.0416	0.0415	0.0406
$A_{2}$	0.3727	0.7000	15.9922	0.0345	0.0332	0.0308	0.0272	0.0211
$A_{3}$	0.3727	0.7000	15.9922	0.0309	0.0287	0.0254	0.0211	0.0141
$A_{4}$	0.3727	0.7000	15.9922	0.0263	0.0231	0.0193	0.0146	0.0081
$A_{5}$	0.3727	0.7000	15.9922	0.0175	0.0145	0.0111	0.0073	0.0031

**Table 7:** Ablation results for the adjacent-swap experiment.

Method	$d(A_{1})$	$d(A_{2})$	$d(A_{3})$	$d(A_{4})$	$d(A_{5})$	$R_{\mathrm{pos}}$
MEOWA-KTC	0.0415	0.0272	0.0211	0.0146	0.0073	0.8234
Uniform-KTC	0.0400	0.0345	0.0309	0.0263	0.0175	0.5624
MEOWA-Jaccard	0.0341	0.0203	0.0131	0.0087	0.0058	0.8286
Uniform-Jaccard	0.0266	0.0217	0.0188	0.0168	0.0154	0.4204

**Table 8:** The RPS-based radar reports.

	$\left(\theta_{1}\right)$	$\left(\theta_{2}\right)$	$\left(\theta_{3}\right)$	$\left(\theta_{1},\theta_{2}\right)$	$\left(\theta_{2},\theta_{1}\right)$	$\left(\theta_{1},\theta_{3}\right)$
$RPS_{1}$	0.04	0.04	0.04	0.24	0	0
$RPS_{2}$	0.09	0.27	0.03	0.02	0.11	0
$RPS_{3}$	0.64	0	0	0.12	0.11	0
	$\left(\theta_{3},\theta_{1}\right)$	$\left(\theta_{1},\theta_{2},\theta_{3}\right)$	$\left(\theta_{1},\theta_{3},\theta_{2}\right)$	$\left(\theta_{2},\theta_{1},\theta_{3}\right)$	$\left(\theta_{2},\theta_{3},\theta_{1}\right)$	$\left(\theta_{3},\theta_{1},\theta_{2}\right)$
$RPS_{1}$	0	0	0.64	0	0	0
$RPS_{2}$	0	0	0.02	0.27	0.14	0.05
$RPS_{3}$	0	0.03	0.04	0.03	0.03	0

**Table 9:** Comparison of RTD values across different methods.

		Comparison Methods			MEOWA-KTC				
Type	Threat Event	Traditional $d_{\mathrm{Chen}}$	PJS	SRP	α=0.5	α=0.6	α=0.7	α=0.8	α=0.9
Type-1	$RTD(\theta_{1})$	**0.7442**	**0.7605**	**0.7224**	**0.7560**	**0.7591**	**0.7622**	**0.7652**	**0.7686**
	$RTD(\theta_{2})$	0.2343	0.2204	0.2536	0.2229	0.2199	0.2170	0.2140	0.2108
	$RTD(\theta_{3})$	0.0215	0.0191	0.0240	0.0211	0.0210	0.0208	0.0207	0.0206
	Decision	$\theta_{1}$	$\theta_{1}$	$\theta_{1}$	$\theta_{1}$	$\theta_{1}$	$\theta_{1}$	$\theta_{1}$	$\theta_{1}$
Type-2	$RTD(\left(\theta_{1},\theta_{2}\right))$	**0.6239**	**0.6084**	**0.6313**	**0.6354**	**0.6385**	**0.6415**	**0.6446**	**0.6478**
	$RTD(\left(\theta_{2},\theta_{1}\right))$	0.3761	0.3916	0.3687	0.3646	0.3615	0.3585	0.3554	0.3522
	$RTD(\left(\theta_{1},\theta_{3}\right))$	0.0000	0.0000	0.0000	0.0000	0.0000	0.0000	0.0000	0.0000
	$RTD(\left(\theta_{3},\theta_{1}\right))$	0.0000	0.0000	0.0000	0.0000	0.0000	0.0000	0.0000	0.0000
	Decision	$\left(\theta_{1},\theta_{2}\right)$	$\left(\theta_{1},\theta_{2}\right)$	$\left(\theta_{1},\theta_{2}\right)$	$\left(\theta_{1},\theta_{2}\right)$	$\left(\theta_{1},\theta_{2}\right)$	$\left(\theta_{1},\theta_{2}\right)$	$\left(\theta_{1},\theta_{2}\right)$	$\left(\theta_{1},\theta_{2}\right)$
Type-3	$RTD(\left(\theta_{1},\theta_{2},\theta_{3}\right))$	0.0245	0.0270	0.0221	0.0252	0.0254	0.0255	0.0257	0.0259
	$RTD(\left(\theta_{1},\theta_{3},\theta_{2}\right))$	**0.5567**	**0.5368**	**0.5663**	**0.5708**	**0.5747**	**0.5784**	**0.5821**	**0.5861**
	$RTD(\left(\theta_{2},\theta_{1},\theta_{3}\right))$	0.2416	0.2513	0.2377	0.2327	0.2304	0.2280	0.2257	0.2232
	$RTD(\left(\theta_{2},\theta_{3},\theta_{1}\right))$	0.1371	0.1433	0.1339	0.1328	0.1317	0.1305	0.1294	0.1282
	$RTD(\left(\theta_{3},\theta_{1},\theta_{2}\right))$	0.0402	0.0416	0.0399	0.0384	0.0380	0.0375	0.0370	0.0365
	Decision	$\left(\theta_{1},\theta_{3},\theta_{2}\right)$	$\left(\theta_{1},\theta_{3},\theta_{2}\right)$	$\left(\theta_{1},\theta_{3},\theta_{2}\right)$	$\left(\theta_{1},\theta_{3},\theta_{2}\right)$	$\left(\theta_{1},\theta_{3},\theta_{2}\right)$	$\left(\theta_{1},\theta_{3},\theta_{2}\right)$	$\left(\theta_{1},\theta_{3},\theta_{2}\right)$	$\left(\theta_{1},\theta_{3},\theta_{2}\right)$

Note: The bold numbers denote the maximum RTD values for each type of threat event.

**Table 10:** Decision margins of different fusion methods and MEOWA-KTC settings.

Method and Setting	Type-1	Type-2	Type-3
Traditional $d_{Chen}$	0.5098	0.2478	0.3151
PJS	0.5401	0.2168	0.2855
SRP	0.4688	0.2625	0.3286
MEOWA-KTC (α=0.5)	0.5331	0.2708	0.3381
MEOWA-KTC (α=0.6)	0.5392	0.2770	0.3443
MEOWA-KTC (α=0.7)	0.5452	0.2831	0.3503
MEOWA-KTC (α=0.8)	0.5512	0.2891	0.3564
MEOWA-KTC (α=0.9)	0.5577	0.2956	0.3629

**Table 11:** Robustness results at the maximum tested settings η=0.30 and ρ=0.30.

Noise Type	Method	Decision Stability	Decision Margin (Mean ± SD)
Mass distribution	MEOWA-KTC (α=0.8)	1.00/1.00/1.00	0.3926 ± 0.0669/0.1490 ± 0.0446/0.2400 ± 0.0417
	Traditional $d_{Chen}$	1.00/1.00/1.00	0.3715 ± 0.0681/0.1346 ± 0.0440/0.2224 ± 0.0411
	PJS	1.00/1.00/1.00	0.3872 ± 0.0764/0.1134 ± 0.0459/0.1952 ± 0.0469
	SRP	1.00/1.00/1.00	0.3869 ± 0.0849/0.1257 ± 0.0515/0.2104 ± 0.0543
Permutation order	MEOWA-KTC (α=0.8)	1.00/0.95/0.79	0.5300 ± 0.0176/0.2746 ± 0.1751/0.3314 ± 0.0752
	Traditional $d_{Chen}$	1.00/0.95/0.79	0.4867 ± 0.0188/0.2517 ± 0.1776/0.2981 ± 0.0793
	PJS	1.00/0.92/0.79	0.5048 ± 0.0401/0.2368 ± 0.1833/0.2782 ± 0.0938
	SRP	1.00/0.95/0.79	0.4850 ± 0.0464/0.2569 ± 0.1841/0.3064 ± 0.0901

Note: Each triplet is ordered as Type-1/Type-2/Type-3.

## Data Availability

The original contributions presented in this study are included in the article. Further inquiries can be directed to the corresponding author.
